# Supplementary material for: Cost-effectiveness of monthly follow-up for the treatment of uncomplicated severe acute malnutrition: An economic evaluation of a randomized controlled trial
Source: PLOS Glob Public Health. 2022 Dec 9;2(12):e0001189. doi: 10.1371/journal.pgph.0001189 (PMC10022243; doi:10.1371/journal.pgph.0001189)
Supplement: S1 Table — (DOCX) [file pgph.0001189.s002.docx]

## **Table S1: Effect of monthly schedule of follow-up compared to standard weekly schedule of follow-up on primary and secondary effectiveness outcomes assessed at program discharge (reproduced from Table 2, Hitchings et al [1]).**


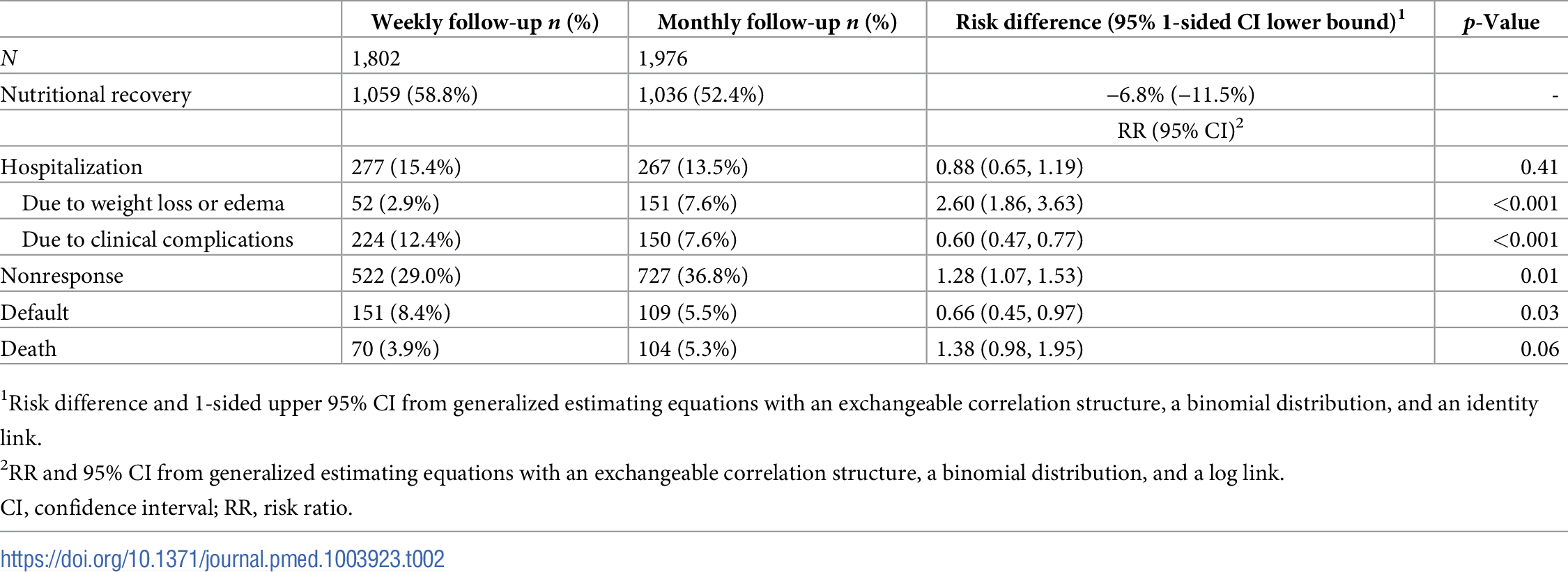


[1] Hitchings MDT, Berthé F, Aruna P, Shehu I, Hamza MA, Nanama S, et al. Effectiveness of a monthly schedule of follow-up for the treatment of uncomplicated severe acute malnutrition in Sokoto, Nigeria: A cluster randomized crossover trial. *PLoS Med*. 2022;19(3):e1003923
