# Supplementary material for: Cost-effectiveness of monthly follow-up for the treatment of uncomplicated severe acute malnutrition: An economic evaluation of a randomized controlled trial
Source: PLOS Glob Public Health. 2022 Dec 9;2(12):e0001189. doi: 10.1371/journal.pgph.0001189 (PMC10022243; doi:10.1371/journal.pgph.0001189)
Supplement: S2 Protocol — (PDF) [file pgph.0001189.s004.pdf]

Reducing the frequency of follow up and task sharing in the treatment of uncomplicated severe acute malnutrition: an evaluation of monthly visits and home-based surveillance for access-limited and high-burden settings

COST-EFFECTIVENESS PROTOCOL

November 15, 2017

1. Background and rationale

The current approach for treating severe acute malnutrition (SAM) in low resource settings combines outpatient treatment with ready-to-use therapeutic foods (RUTF) for uncomplicated cases and inpatient treatment only for complicated cases. This approach has proven both efficient and effective compared to earlier approaches (routine hospitalization for all SAM cases), however is still difficult to implement in some settings, with the frequent OTP visits required for outpatient care placing burdens on both service providers and patient caregivers.

This trial tests different approaches for implementing community-based treatment for SAM. The current approach, which requires weekly follow-up of patients receiving outpatient care, is compared to a revised approach involving monthly outpatient follow up. The details of the trial are described in the overall study protocol *("Reducing the frequency of follow up and task sharing in the treatment of uncomplicated severe acute malnutrition: an evaluation of monthly visits and home-based surveillance for access-limited and high-burden settings")*. This cost-effectiveness protocol describes the design, data collection, and analysis plan for the economic component of this trial, which is designed to estimate the cost effectiveness of the new approach (with a reduced schedule of follow up) compared to the current approach.

2. Objectives and endpoints for cost effectiveness analysis

The objective of the economic component of the trial is to assess the cost-effectiveness of monthly vs. standard weekly follow up in the treatment of uncomplicated severe acute malnutrition, in children aged 6 to 59 months.

The primary cost-effectiveness endpoint will be the incremental cost per child achieving nutritional recovery under the reduced follow-up approach, as compared to the standard approach. Secondary cost-effective end-points will include (i) the average and incremental cost per child treated, (ii) the incremental cost per death averted, (iii) the incremental cost per life year saved, and (iv) the incremental cost per Disability Adjusted Life Year (DALY) averted, for the revised approach as compared to the current approach. The first of these secondary endpoints will describe overall resource consumption associated with each intervention strategy, which can be useful information for program planning/budgeting. The last three secondary endpoints provide information on the efficiency of the reduced follow-up strategy in improving summary measures of population health, and allow comparison to other health interventions.

3. Evaluation framework

3.1. Population

The population of interest is children 6-59 months of age, who are newly admitted for the treatment of uncomplicated SAM at outpatient therapeutic feeding centers. This

includes both previously untreated cases and relapse cases, and excludes cases requiring immediate inpatient care following clinical evaluation and returned defaulters.

3.2. Strategies to be compared

The cost effectiveness analysis will compare two strategies, matching those tested in the parent trial:

1) Standard weekly visits at the outpatient therapeutic feeding center until discharge (***standard approach***).

2) Monthly visits at the outpatient therapeutic feeding center with caregiver support for home-based surveillance, with visits scheduled at weeks 4, 8, 10 and 12 until discharge (***reduced follow-up approach***). As compared to the standard approach, caregivers receive additional instruction and take home materials regarding home-based MUAC and clinical surveillance at their admission appointment.

Under both strategies, children receive standard medical care as per national guidelines. Regularly scheduled follow-up visits include a rapid health assessment and weight check by a nurse, and a physical exam if signs of illness or weight loss are present. Caregivers are instructed to return to the health center between scheduled follow up visits if any clinical sign of concern develops. Cost-effectiveness analyses will be conducted from an intention-to-treat approach.[1]

3.3. Costing perspective

For the main analysis we will assess costs from the societal perspective, which includes costs incurred by both providers and recipients of health services. Provider costs will include staffing (salaries, per diems, and volunteer incentives), capital costs (building space, equipment), consumables (medical supplies, medications), and overhead costs (electricity, water, and maintenance). We will collect costs incurred at the OTP-level (providing services to children and caregivers), as well as at the coordination center (e.g. training, monitoring and supervision), in order to estimate the full costs of providing outpatient SAM care. Patient costs will include direct medical costs (medicines and other medical consumables or services paid for by participants relating to SAM treatment), non-medical costs (participant-incurred expenses for travel, food, lodging, and child care relating to SAM treatment), and the opportunity cost of participant time spent seeking/receiving SAM care. Costs of research-related activities (data collection, study coordination) will be excluded. Prices will be based on those observed in the trial.

In sensitivity analyses we will calculate costs under a provider perspective (excluding patient costs / cost savings), and with national program price levels (where these differ from values observed in the trial).

3.4. Analytic horizon and time frame

Endpoints for the cost per child recovered, cost per child treated, and cost per death averted will consider costs and health outcomes observed from trial entry until the 2-month post-discharge follow-up visit. For the cost per life year saved and cost per DALY averted endpoints, analyses will consider all outcomes until death.

The timeframe for empirical data collection will be the same as for the parent trial. Outcomes realized after the 2-month post-discharge follow-up visit will be estimated using standard decision analytic techniques[2].

3.5. Coverage changes

In the main cost-effectiveness analysis the comparison between standard and reduced follow-up strategies will be based on differences in outcomes for individuals enrolling in the trial under either arm. This approach ignores the potential for differences in coverage achieved by the different strategies (ie differential effectiveness in enrolling patients). In sensitivity analyses we will recalculate cost-effectiveness results allowing for differences in coverage between study arms.

4. Data collection

4.1. Health outcomes

Details of data collection for health outcomes are described in the main study protocol for the effectiveness trial. Data required to assess health outcomes for the cost-effectiveness analysis will include patient–level data on (1) whether the individual achieved nutritional recovery (binary, required for the primary cost-effectiveness endpoint), and (2) final health outcome recorded (recovery/death/default, required for estimating DALYs averted). Group-level coverage data (based on coverage surveys conducted before and after the trial) will also be used for a sensitivity analysis on coverage differences. Individual-level data on health status at trial entry will be used in sensitivity analyses controlling for differences in case-mix between study arms.

Additional evidence required to calculate DALYs averted will be drawn from the published literature[3] and routine databases.[4]

4.2. Provider costs

Provider costs will include staffing (salaries, per diems, and volunteer incentives), capital costs (vehicles, equipment), consumables (medical supplies, medications), and overhead costs (building space, electricity, water, and maintenance, and intervention-specific training for study staff to provide caregiver education). We will include the costs of all activities undertaken to provide the intervention, even if some of these activities might not be done (or done less frequently) in the routine national program. We will exclude the cost of all activities undertaken solely for research purposes (e.g. consenting participants, collecting baseline/background data, etc).

4.2.1. Unit costs

Provider cost data will be used to estimate unit costs for a standardized set of clinical services (Table 1).

Table 1: Clinical service definitions

Clinical service	Units	Definition	Costs included
Education session	Per individual trained	Education provided to caregivers at treatment enrollment (reduced follow-up arm)	Staff time, educational materials, management and infrastructure overheads including staff training
Therapeutic food	Per sachet provided	Sachets of therapeutic food provided to study participants	Therapeutic food sachets, including supply chain costs (customs, transport, storage, etc)
Enrollment visit	Per visit	Initial outpatient visit, enrollment in SAM care	Staff time, management and infrastructure overheads
Follow-up OTP visits	Per visit, stratified by services received	Scheduled and unscheduled outpatient follow-up visits related to SAM treatment, stratified by uncomplicated/complicated.	Staff time, management and infrastructure overheads
Home visits	Per visit	Home visits for spot checks, home-based surveillance, and home visits for tracking of defaulters	Volunteer time, staff time, transport, management overheads
Inpatient days*	Per day	Inpatient SAM care, provided to children initially screened to receive outpatient care	Hospital hotel costs, infrastructure/mgmt overheads, medical supplies and services

* The costs of inpatient care are included because the intervention may lead to changes in the probability and length of inpatient care received by children with SAM, even though they are treated in an outpatient setting.

For education sessions and OTP visits, unit costs will be calculated by summing resource use under each budget category, using a macrocosting approach. Staff time will be estimated by asking each staff member engaged in providing SAM care to allocate their time across a set of activity categories: SAM caregiver education sessions, SAM OTP visits, organization of home visits, and other non-clinical activities. Time allocated to 'other non-clinical activities' will be treated as an overhead, and allocated proportionally over the other activity categories. For each clinical staff member, the FTE spent providing each clinical service will be multiplied by their loaded salary, and the result summed across staff members to calculate the total staffing costs for each clinical service (caregiver education sessions, OTP visits) at an OTP level, which will then be divided by the number of each service provided by the OTP to calculate the unit cost. Costs for consumables (training supplies etc.) will be calculated using a similar approach, by dividing total consumption of each item (including wastage) by the number of caregiver education sessions or OTP visits reported by the clinic.

For therapeutic food, unit costs will be based on purchase prices, plus supply chain costs (customs, transport, storage).

For home visits (conducted by volunteers and staff), costs will be calculated by dividing total staff costs (stipends made to study volunteers plus staff time multiplied by their loaded salary) by the total number of home visits reported.

For hospital bed-days, cost data will be collected for the two district hospitals (stabilization centers) supporting the study sites, to calculate the total costs associated with providing inpatient services, which will be divided by total bed-days to calculate the average unit cost across both hospitals.

4.2.2. Management, administration and infrastructure at OTP-level

The costs of management, administration, and infrastructure will be calculated at an OTP level, via direct allocation. That is, all expenses for management, administration and infrastructure incurred at the OTP will be estimated as a fraction of total expenses, then allocated proportionally to the unit costs described in Table 1.

4.2.3. Costs incurred at the coordinating center-level

We will collect costs on activities undertaken at the coordination center to support the OTPs. This will include any training / refresher training of health workers, supervision, M&E, and other management or support tasks. To calculate these costs, the total costs incurred to support OTPs (including the study sites as well as other sites supported by the coordination center) will be summed for the 12 month study period, then divided by the total number of children treated over that period to calculate a per-child cost.

4.2.4. Unit volume

For each study participant, information on the number of clinical services received (according to the categories described in Table 1) will be recorded routinely as part of data collection for the parent trial.

4.3. Patient costs

Patient costs will be collected in order to calculate costs under a societal perspective. Patient costs will be collected through two questionnaires administered to a subsample of caregivers of SAM patients. Average patient costs for each participant will be calculated by summing direct medical, non-medical, and indirect costs reported by each participant.

5. Statistical analysis

5.1. Estimation of economic costs

To calculate economic costs, investments will be annualized over their useful life using standard methods, assuming a 3% discount rate.[5] Prices will be based on those observed

during the trial. Prices for items purchased in past years will be inflated to current values, and results will be reported in current US dollars and local currency units.

5.2. Estimation of total per-patient costs

For an individual study participant, total provider costs will be calculated by multiplying unit costs for each clinical service by the number of clinical services received, then summing across all clinical service categories. Total patient cost will be calculated by summing the costs reported in patient questionnaires (Costing Forms B and C).

5.3. Estimation of long-term health outcomes

To estimate DALYs averted, estimates of Disability Adjusted Life Expectancy will be calculated based on final health status observed in the trial.[6] Life expectancy conditional on survival to the end of the trial will be based on population life expectancy estimates at age of discharge.[4] Disability weights will be based on published values.[3] Outcomes realized in future years will be discounted at 3% per year.[5] Estimates of DALYs averted will ignore differences in health burden associated with differences in time to nutritional recovery in the trial, as these differences will be trivial compared to reductions in DALYS from averted morality.

In sensitivity analyses we will calculate cost-effectiveness results allowing for differences in coverage between intervention strategies, using data from coverage surveys conducted before and after the trial. To implement this analysis we will need an estimate of the cost and health outcomes realized by individuals with uncomplicated SAM who are not successfully enrolled. As empirical information on this group will not be collected in the trial (apart from an estimate of coverage achieved), these unobserved outcomes will be estimated from the published literature.

5.4. Missing data

Missing data for the cost-effectiveness analysis may arise in the context of patient default, or if caregivers decline to provide information on costs borne to receive care. If missingness is minor (<5%) we will conduct a complete case analysis. If missingness is more extensive we will use standard techniques to impute missing data.[7,8]

5.5. Cost-effectiveness outcomes

Primary cost-effectiveness endpoints will be reported as incremental cost-effectiveness ratios (ICERs), defined as the arithmetic mean difference in costs for the reduced follow-up approach compared to the standard approach divided by the arithmetic mean difference in health outcomes achieved by the reduced follow-up approach compared to the standard approach.[5] Given that the number of patients enrolled in each study arm may differ, both costs and health outcomes will first be normalized to a per-patient basis by dividing by the number of patients initiating treatment under each study arm. As a consequence, the ICER for the primary study endpoint will be equal to the arithmetic mean per-patient cost between study arms divided by the arithmetic mean difference in the probability of recovery between study arms.

In the instance that uncertainty in incremental costs and/or health outcomes covers multiple quadrants of the cost-effectiveness plan, incremental cost effectiveness ratios and interval measures of uncertainty can be misleading. Such a situation may occur in this trial, where the reduced follow-up strategy may be cost-saving and have incremental health outcomes near to zero, as compared to the current strategy. In this situation Net Monetary Benefit,[9] which is not affected by these issues, will be calculated as an additional cost-effectiveness outcome.

5.6. Uncertainty analysis

For all cost-effectiveness outcomes, measures of uncertainty will be calculated by via a non-parametric bootstrap[1] of the participant-level estimates of cost and effectiveness endpoints (taking account of the clinic-level clustering), with results presented as confidence intervals (as appropriate) and cost-effectiveness acceptability curves (CEACs). Where calculation of costs and/or health outcomes involve additional assumptions around which there is some uncertainty (e.g. useful life for equipment when calculating capital costs, or life expectancy estimates when calculating DALYs averted), this uncertainty will be incorporated into the analysis through a 2nd-order Monte Carlo simulation: (i) probability distributions will parameterized to represent the uncertainty in each parameter, (ii) for each bootstrap replicate a random value will be drawn from each parameter distribution and results calculated, (iii) outcomes (ICERs, confidence intervals, CEACs) will be calculated from the distribution of these results.[10] In addition to these measures of global uncertainty, univariate sensitivity analyses will be conducted to assess the robustness of cost-effectiveness results to uncertainty in individual costs and other determinants of intervention efficiency.

5.7. Additional sensitivity analyses

In sensitivity analyses we will examine the sensitivity of cost-effectiveness results to differences in case-mix between study arms. Children can meet intervention enrollment criteria with MUAC < 115 mm and/or grade 1-2 edema. Differences in the health status of patients at enrollment could lead to differences in the probabilities of outcomes relevant to the cost effectiveness analysis (death, nutritional recovery, hospitalization). For these reasons we will recalculate cost-effectiveness results controlling for case-mix. This will be achieved by stratifying the enrolled population by eligibility category, and reweighting results for strata within each study arm to reproduce the case-mix distribution in the overall trial population. We will also conduct sensitivity analysis to test the effect of using input prices from the national program.

6. Citations

1. Ramsey SD, Willke RJ, Glick H, et al. Cost-effectiveness analysis alongside clinical trials II-An ISPOR Good Research Practices Task Force report. *Value Health* 2015; **18**(2): 161-72.
2. Siebert U, Alagoz O, Bayoumi AM, et al. State-transition modeling: a report of the ISPOR-SMDM Modeling Good Research Practices Task Force-3. *Med Decis Making* 2012; **32**(5): 690-700.
3. Salomon JA, Vos T, Hogan DR, et al. Common values in assessing health outcomes from disease and injury: disability weights measurement study for the Global Burden of Disease Study 2010. *Lancet* 2012; **380**(9859): 2129-43.
4. United Nations Population Division. World Population Prospects: The 2010 Revision, CD-ROM Edition. Geneva: United Nations, Department of Economic and Social Affairs, Population Division.; 2011.
5. Sanders GD, Neumann PJ, Basu A, et al. Recommendations for Conduct, Methodological Practices, and Reporting of Cost-effectiveness Analyses: Second Panel on Cost-Effectiveness in Health and Medicine. *JAMA* 2016; **316**(10): 1093-103. doi: 10.01/jama.2016.12195.
6. Hutubessy R, Chisholm D, Edejer TT. Generalized cost-effectiveness analysis for national-level priority-setting in the health sector. *Cost Eff Resour Alloc* 2003; **1**(1): 8-.
7. Burton A, Billingham LJ, Bryan S. Cost-effectiveness in clinical trials: using multiple imputation to deal with incomplete cost data. *Clinical trials (London, England)* 2007; **4**(2): 154-61.
8. Gomes M, Diaz-Ordaz K, Grieve R, Kenward MG. Multiple imputation methods for handling missing data in cost-effectiveness analyses that use data from hierarchical studies: an application to cluster randomized trials. *Med Decis Making* 2013; **33**(8): 1051-63.
9. Stinnett AA, Mullahy J. Net health benefits: a new framework for the analysis of uncertainty in cost-effectiveness analysis. *Med Decis Making* 1998; **18**(2 Suppl): S68-80.
10. Briggs AH, Weinstein MC, Fenwick EA, Karnon J, Sculpher MJ, Paltiel AD. Model parameter estimation and uncertainty analysis: a report of the ISPOR-SMDM Modeling Good Research Practices Task Force Working Group-6. *Med Decis Making* 2012; **32**(5): 722-32.

7. Appendix: data collection forms

Data to estimate costs will be collected with the following forms:

Form 1: Outpatient Therapeutic Program (OTP) card
Form 11: Exit form

A. Educational session time register
B. Patient outpatient cost questionnaire
C. Patient hospital cost questionnaire
D. OTP and hospital cost spreadsheet

Form 1, Form 11 and Costing Form A will collect information on the time spent by patients and caregivers waiting to receive outpatient SAM care, and the time spent by OTP staff providing care. The patient cost questionnaires (Costing Forms B and C) will collect information on out-of-pocket expenses incurred by caregivers during the course of SAM care and caregiver education. Costing Form B will be used for outpatient care, and Costing Form C will be used for hospital-based care. The final data collection tool (Costing Form D) will collect information on health facility resources (OTP, hospital) used to provide care for SAM patients.

A. Form 1 (Clinical care time)

This form will collect information on the total time spent by patients and caregivers attending the outpatient SAM treatment clinic. The following variables are included:

- Time of arrival (caregiver and patient check in)
- Time of departure (caregiver and patient depart clinic)

B. Form 1 (Clinical care time)

This form will collect information on the time spent by OTP staff providing care to outpatient SAM patients. The following variables are included:
- Start time of consultation (caregiver/child asked to enter consultation area)
- End time of consultation (caregiver/child exit consultation area, paperwork complete, area prepared for next patient)

C. Costing Form A: Educational session register

This form will collect information on the time spent by OTP staff providing the educational session for caregivers of SAM patients at their initial visit (only applies to intervention arm). The following variables are included:
- Date

- Clinic/OTP ID
- Start time of educational session (begin preparing for training)
- End time of educational session (all caregivers have departed from training)
- Number of caregivers trained.

D. Costing Form B: Patient outpatient cost questionnaire

This form will collect information on out-of-pocket expenses incurred by caregivers during the course of outpatient SAM care, and at the 2-month follow-up visit after discharge. The following variables are included:
- Date
- Clinic/OTP ID
- Patient ID
- Expenditures on travel, food, accommodation, childcare, medicines, and any other costs related to the current OTP visit.
- Expenditures on travel, food, accommodation, childcare, medicines, procedures, and any other costs related to care received since the last OTP visit before the current one.

Data will be collected from a subsample of caregivers and patients enrolled in the overall trial ('outpatient costing subsample'). The first five patients enrolled into the trial at each site during the 12 costing weeks (Figure S1) will be included in the outpatient costing subsample, for a total of 60 patients per site and 600 patients total.

Figure S1: Timing of cost data collection ('costing weeks').

JULY 2017

m	t	w	t	F	s	s
					1	2
4	4	5	6	7	8	9
10	11	12	13	14	15	16
17	18	19	20	21	22	23
24	25	26	27	28	29	30
31						

AUGUST 2017

m	t	w	t	f	s	s
	1	2	3	4	5	6
7	8	9	10	11	12	13
14	15	16	17	18	19	20
21	22	23	24	25	26	27
28	29	30	31			

SEPTEMBER 2017

m	t	w	t	f	s	s
				1	2	3
4	5	6	7	8	9	10
11	12	13	14	15	16	17
18	19	20	21	22	23	24
25	26	27	28	29	30	

OCTOBER 2017

M	t	w	t	f	s	s
						1
2	3	4	5	6	7	8
9	10	11	12	13	14	15
16	17	18	19	20	21	22
23	24	25	26	27	28	29
30	31					

NOVEMBER 2017

m	t	w	t	F	s	s
		1	2	3	4	5
6	7	8	9	10	11	12
13	14	15	16	17	18	19
20	21	22	23	24	25	26
27	28	29	30			

DECEMBER 2017

m	t	w	t	f	s	s
				1	2	3
4	5	6	7	8	9	10
11	12	13	14	15	16	17
18	19	20	21	22	23	24
25	26	27	28	29	30	31

JANUARY 2018

m	t	w	t	f	s	s
1	2	3	5	5	6	7
8	9	10	11	12	13	14
15	16	17	18	19	20	21
22	23	24	25	26	27	28
29	30	31				

FEBRUARY 2018

M	t	w	t	f	s	s
			1	2	3	4
5	6	7	8	9	10	11
12	13	14	15	16	17	18
19	20	21	22	23	24	25
26	27	28				

MARCH 2018

m	t	w	t	F	s	s
			1	2	3	4

APRIL 2018

m	t	w	t	f	s	s
						1

MAY 2018

m	t	w	t	f	s	s
	1	2	3	4	5	6

JUNE 2018

M	t	w	t	f	s	s
				1	2	3

5	6	7	8	9	10	11	2	3	4	5	6	7	8	7	8	9	10	11	12	13	4	5	6	7	8	9	10
12	13	14	15	16	17	18	9	10	11	12	13	14	15	14	15	16	17	18	19	20	11	12	13	14	15	16	17
19	20	21	22	23	24	25	16	17	18	19	20	21	22	21	22	23	24	25	26	27	18	19	20	21	22	23	24
26	27	28	29	30	31		23	24	25	26	27	28	29	28	29	30	31				25	26	27	28	29	30	
							30																				

For the patients enrolled in the outpatient costing subsample, data will be collected via Costing Form B at all scheduled follow up OTP visits the patient attends until discharge. Costing Form B will be filled out one final time at the 2-month follow-up visit after discharge.

E. Costing Form C: Patient hospital cost questionnaire

This form will collect information on out-of-pocket expenses incurred by caregivers during the course of hospitalization for SAM care at a stabilization center (SC). The following variables are included:

- Date
- Clinic/OTP ID
- Patient ID
- Total days spent at hospital (e.g. length of SC stay)
- Expenditures on transport to/from the hospital.
- Expenditures on food during the hospitalization.
- Expenditures on accommodation during the hospitalization.
- Expenditures on childcare during the hospitalization.
- Expenditures on medicines during the hospitalization.
- Expenditures on tests and procedures during the hospitalization.
- Other expenses incurred during the hospitalization.

Data will be collected for all SAM patients that have been hospitalized using Costing Form C on a weekly basis and at discharge from the SC. All children hospitalized with SAM are eligible for inclusion, including those admitted directly to SC and those who were admitted first to OTP and then transferred for a medical complication.

F. Costing Form D: OTP and hospital cost spreadsheet

This form will be completed by the project administrator at the completion of the study, and collect information on facility resources (OTP, hospital) used to provide care for SAM patients, and coordination center resources used to support these activities. The Excel spreadsheet (Costing Form D: OTP and hospital cost spreadsheet) consists of a set of spreadsheet pages, one for each budget category, including staff salaries, volunteer stipends, therapeutic food, other supplies, vehicles and equipment, utilities, buildings, and intervention-specific training. Data for this costing form will be drawn from study records and will not require field data collection. Data on expenditures during the study

period, as well as infrastructure and equipment used during the study period, are to be entered for each study OTP as well as for the hospitals and coordination center.
