# Supplementary material for: Cost-effectiveness of monthly follow-up for the treatment of uncomplicated severe acute malnutrition: An economic evaluation of a randomized controlled trial
Source: PLOS Glob Public Health. 2022 Dec 9;2(12):e0001189. doi: 10.1371/journal.pgph.0001189 (PMC10022243; doi:10.1371/journal.pgph.0001189)
Supplement: Attachment — Submitted filename: CIFF CEA_PGPH itemized response 2022.09.23.pdf [file pgph.0001189.s005.pdf]

Itemized response to reviewer comments

"Cost-effectiveness of monthly follow-up for the treatment of uncomplicated severe acute malnutrition: an economic evaluation alongside a randomized controlled trial"

(PGPH-D-22-00813)

Thank you for the opportunity to revise our manuscript. Below we provide itemized responses to the editor's and reviewers' comments. We have renumbered these for the purpose of this response.

Editor's comments

1. In the online submission form, you indicated that "The deidentified dataset supporting this research can be made available following a submitted request as per Epicentre and General Data Protection Regulation (EU) 2016/679 data sharing policy." All PLOS journals now require all data underlying the findings described in their manuscript to be freely available to other researchers, either 1. In a public repository, 2. Within the manuscript itself, or 3. Uploaded as supplementary information. This policy applies to all data except where public deposition would breach compliance with the protocol approved by your research ethics board. If your data cannot be made publicly available for ethical or legal reasons (e.g., public availability would compromise patient privacy), please explain your reasons by return email and your exemption request will be escalated to the editor for approval. Your exemption request will be handled independently and will not hold up the peer review process, but will need to be resolved should your manuscript be accepted for publication. One of the Editorial team will then be in touch if there are any issues.

Authors' response: We are unable to share data underlying the findings described in this manuscript due to General Data Protection Regulation regulations. All requests for these data must be compliant with per Epicentre and General Data Protection Regulation (EU) 2016/679 data sharing policy. For this manuscript we have proposed the same data sharing approach as used for the primary analysis of this study, also published in a PLoS journal (Hitchings MDT et al, PLoS Med 2022). As requested, we have submitted a request for exemption to the editor by return email.

2. Thank you for submitting your paper to PGPH for publication. Two independent reviewers have assessed your manuscript and found it to have merit. However, they raised substantial methodological issues that will need your attention.

   The issues include:

   - Clarity on the quantity of therapeutic food per participant, and the extended period of visits.
   - They also felt there is a need for you to look at the definition of nutritional recovery.
   - The stage at which participants were discharged.
   - Justification of sample size used in the study.
   - The assumptions made in the sample size estimation.
   - There is also a need for clarity on the presentation of the results.

Authors' response: Thank you for these comments. We have responded to each of these issues raised by the reviewers, as detailed below.

Reviewer #1

3. This paper presents an economic evaluation of monthly vs weekly follow-up treatment for malnutrition, based on the findings of a recent randomized controlled trial. Understanding the cost tradeoffs in malnutrition treatment and prevention is an important topic, and this paper provides valuable, and somewhat counter-intuitive evidence on an aspect of great importance in operating a malnutrition treatment program, namely that monthly follow-up produced worse outcomes at a higher cost than weekly followup.

Authors' response: Thank you for these kind words.

4. There is one major methodological issue which I would like to better understand before I can determine the validity of this work, however. On line 158, the authors define their outcome of nutritional recovery as being free from complications and with MUAC of at least 125 mm for 2 consecutive visits. But it seems to me that since the treatment variable itself is the time between successive visits, this criteria might inadvertently lead to the treatment arm appearing less efficacious than the control arm, where there are successive visits closer together towards the end of the study. It appears possible, at least in theory, that this requirement could produce a bias the classification of recovery for the monthly treatment arm. As a thought experiment, consider if instead, caregivers had taken at-home measurements and reported MUAC at least 125 mm for two consecutive weeks to define recovery. If there are only three follow-up visits for the monthly follow-up arm, children must have a time-to-recovery of less than 8 weeks to be classified as recovered in this arm but could take up to 11 weeks to recover in the standard-of-care arm. Has this risk been addressed in the RCT in a way that could be explained in this paper?

Authors' response: We thank the reviewer for this important observation and agree with the reviewer that the greater spacing between visits in the intervention arm (e.g. monthly follow up) could indeed result in a longer length of stay in the program before nutritional recovery is declared, compared to the standard care with weekly follow-up. This effect was indeed addressed in the primary analysis of the RCT that examined time to programmatic recovery as a secondary outcome. The primary analysis, as suggested by the reviewer, showed that the average time to programmatic recovery was in fact longer in the monthly follow-up arm, compared to weekly follow-up. As the reviewer notes, this longer time to recovery in the monthly arm may be due to the longer spacing between measurements. We do not consider this as a bias but rather a true representation of the programmatic outcome of interest. We confirm that the differential length of stay in the program noted here is included as a secondary outcome in the primary analysis and is accounted for in the costs of therapeutic foods in this cost effectiveness analysis. For transparency, and to respond to the reviewer's comment, we have also included the table of primary and secondary outcomes from the parent trial (reproduced from Hitchings et al 2022) as a supplementary table (Table S1).

5. Line 362: I find it surprising that monthly approach required so many more sachets of therapeutic food. Your explanation about the extended period between visits is not totally satisfying to me. Is this because there is also a longer time-to-recovery in the monthly treatment arm, given the lower recovery rates, or simply because of wastage after recovery since the family has been supplied with a months worth of food? Is it perhaps not only that more food is distributed, but also that more food is required and/or consumed in the treatment arm of the trial? Is there more wastage or loss, e.g. as the family shares with

relatives and neighbors when they have a month's worth of sachets in their house. Any expansion on theorizing why this surprising finding has happened would be a welcome addition to the discussion.

Authors' response: We thank the reviewer for highlighting this important point for discussion. We confirm that there is a longer time to programmatic recovery in the monthly follow-up arm compared to the standard weekly follow-up, as is reported in the primary analysis of the RCT (Hitchings MDT et al, PLoS Med 2022). We are unable to discern whether children in the monthly follow-up arm achieved anthropometric recovery before programmatic recovery was determined, as measurements were only taken at the health center during scheduled study visits as per the study protocol. We therefore cannot confirm the reviewer's hypothesis of whether children in this monthly arm needed food for longer or not. Findings related to adherence to the intervention is reported in the primary analysis of the RCT, which reports high compliance in both groups (98% compliant in the monthly group vs 99.6% compliant in the weekly group).

6. Line 414: The point about the risk of generalizing these results to other settings (line 380), given the low recovery rates in both study arms seems important enough to include in the conclusion.

Authors' response: Thank you for this suggestion. We have edited the text of the conclusions to highlight this point as follows: "<u>Generalization of</u> these findings <u>should proceed with caution given the contextual factors that may have influenced health outcomes in this single study</u>. Concerns <u>related to</u> the burden that weekly visits place on both caregivers and providers <u>and could potentially</u> be alleviated by monthly follow-up <u>remain valid</u>, but <u>the findings from this study</u> suggest that alternative approaches may be needed to address <u>this issue</u>." [lines 511-516]'

7. Line 374-377: I recommend that you include the costs of training and supervision if you can. Even if they are common to the treatment and control arm, it would be a missed opportunity if readers of your paper could see the scale of these essential parts of a successful program in context. It would be great to also include them in Table 1, for busy readers who might miss them in the text.

Authors' response: We agree with the reviewer that these estimates would be of value to some readers. However, as the study was designed to estimate the outcomes required for an incremental cost-effectiveness analysis, we did not collect data on costs that did not differ between study arms, and therefore cannot report this result.

8. Line 88: typo, should be "enrollment"

Authors' response: Thank you for spotting this, we have corrected this typo.

9. Line 91: Why does monthly follow-up include a visit at 10 weeks? Perhaps "monthly" is a confusing term for this less frequent visit schedule.

Authors' response: We thank the reviewer for highlighting this detail. We confirm that the monthly schedule of follow-up included an additional visit at 10 weeks on the recommendation of the study's advisory committee. It was suggested that following 8 weeks of treatment, it was likely that another full month of treatment may not be needed and that an intermediate visit at 10 weeks may be an efficient option to allow recovered children to be discharged before 12 weeks. In response to the reviewer's comment, we have added quotations around the term monthly where the monthly follow-up intervention is defined to indicate that the label "monthly" in this case is shorthand or a simplification in order to label the intervention (line 96).

10. Line 91: Do these visits always end at 12 weeks? If there is a strict limit on the duration of the treatment, after which point children who are not recovered are still discharged from treatment, I would like this to be described in more detail.

Authors' response: We thank the reviewer for highlighting this omission. We confirm that there was, as per the national protocol, a maximum duration of treatment of 12 weeks after which time a child that did not meet the criteria for recovery could be considered to be non-respondent and transferred to hospital care. In response to the reviewer's comment, we have added the following clarification to the text: "Nutritional recovery (used to report the cost per child recovered) was defined <u>at or before 12 weeks from admission</u> as being free from medical complications, MUAC ≥ 125 mm for 2 consecutive visits, and no edema if admitted with edema at study discharge." [lines 181-183]

11. Line 132: Can you describe in more detail how you determine the average visit duration?

Authors' response: We confirm that the average visit duration was calculated as the difference between the start and end of the clinical consultation. In response to the reviewer's comment, we have edited the text to include this additional precision as follows: "<u>For each visit, the visit duration in minutes was calculated as the difference between the start (caregiver/child enter consultation area) and end (caregiver/child exit consultation area, paperwork is complete, area is prepared for next patient) times of the clinical encounter. The average visit duration was calculated as the sum of these estimates divided by the number of visits</u>". [lines 151-155]

12. Line 159: Please define precisely what it means for a child default.

Authors' response: We have edited the text to add the definition of this outcome: "Children who defaulted <u>(defined as 3 missed scheduled clinic visits in the weekly follow-up group and 1 missed scheduled clinic visit in the monthly follow-up group)</u>..." [lines 182-184]

13. Line 168: Please inform the reader of the time-horizon for the expected mortality after the trial here. (Is it 12 months?)

Authors' response: We thank the reviewer for highlighting this point of confusion. In response to the reviewer's comment, we have revised the text to clarify this point: "Under this approach we considered observed mortality during the trial (up to the 3-month post-discharge visit) as well as expected mortality after the trial <u>until 12 months post-enrollment</u>, which was calculated by imposing different disease-specific mortality rates depending on whether an individual was

classified as recovered by the 3-month post-discharge visit. These were multiplied by life expectancy estimates taken from the Global Burden of Disease Study reference life table (21) (Table 1). All mortality attributable to the SAM episode was assumed to occur in the 12 months following study enrollment." [lines 193-199]

14. Line 565: typo, should be "not conducted" instead of "no conducted".

Authors' response: Thank you for identifying this typo, which we have now corrected.

15. Figure 1A, 1B, and 2A: Consider anchoring axes at zero to contextualize the difference between study arms relative to the absolute magnitude of each study arm --- this might tell a different story about the value of monthly follow-up in a setting where weekly follow-up is simply infeasible.

Authors' response: Thank you for this suggestion. We created this figure for comparison (shown below), but prefer the original version as it provides a clearer visualization of the incremental difference between the study arms. Moreover, we feel that the inclusion of (0,0) in the alternative figure implicitly invites comparison to this intercept, yet this is not an appropriate comparison as there is no realistic scenario in which one should expect zero programmatic recovery and zero costs.

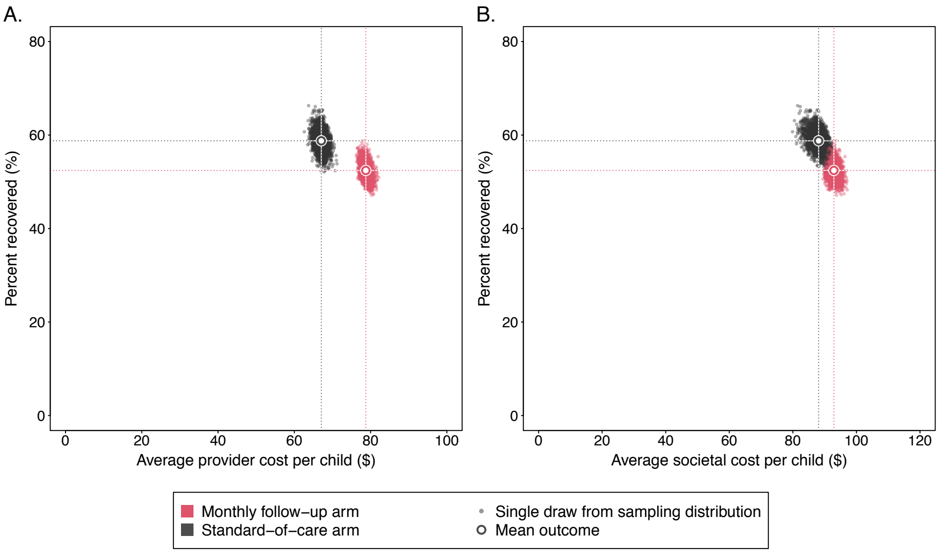

16. Figure 2B: I recommend excluding this figure, since the treatment is never cost-effective.

Authors' response: Thank you for this suggestion. We have edited the figure to remove Panel B (revised version shown below), and edited the text for consistency: "A cost-effectiveness acceptability curve calculated from these results showed that monthly follow-up had <1% probability of being cost-effective for a wide range of willingness-to-pay values, ranging from $10-$10,000 per DALY averted." [lines 368-370]

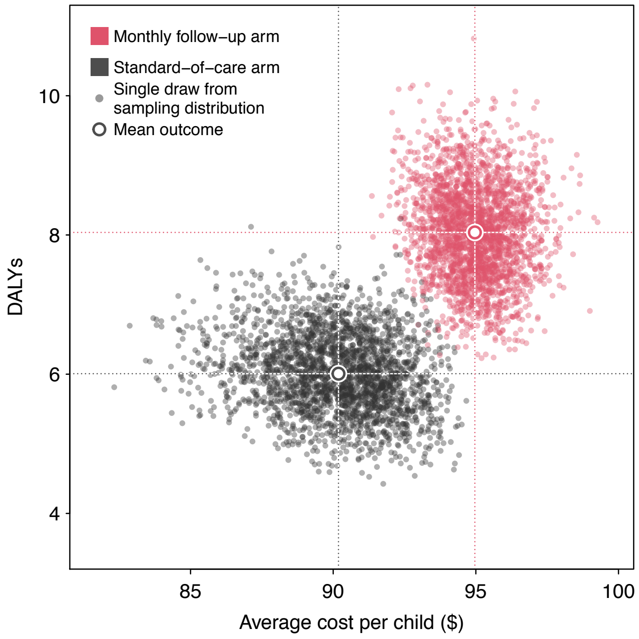

Reviewer #2

17. Overall this is an important paper and well worthy of publication in a high profile journal like PLoS Global Health. It is a well written report of a well done and robustly analysed study.

Authors' response: Thank you for these kind words.

18. I have a few minor comments which I hope will help future readers make the most of the study: it's not very clear (in title, abstract or final sentence of intro), whether this paper presents in-depth economic evaluation of a RCT that's reported elsewhere (as suggested by reference 14 and by line 106) OR whether the cost-effectiveness was the major RCT outcome and this is the main paper reporting all outcomes. Please clarify. If the former, it'd be better to say this was an "economic evaluation OF a (separately reported) RCT". It'd also be good in this case to briefly summarise the main RCT results in the methods. The word "alongside" is what leads to the ambiguity – change that.

Authors' response: Thank you for suggesting this clarification. We have edited the text identified ("alongside") where this appears (title line 2; abstract, line 31; introduction, line 79). We have also added a citation to the publication on the primary effectiveness endpoints earlier in the manuscript (Introduction, line 80) to avoid confusion and orient the reader to the primary results, and included a table of primary and secondary effectiveness outcomes from the parent trial (reproduced from Hitchings et al, 2022) as a supplementary table (Table S1). We have added text to the manuscript noting this: "<u>Table S1 provides summary results for major effectiveness endpoints, as reported in Hitchings et al (15)</u>." [lines 189-190]

19. Consider giving some latest WHO/UNICEF numbers of children affected by SAM worldwide. This would help emphasize the importance of the study to readers not familiar with the field.

Authors' response: We have added this text to the introduction: "In 2020, an estimated 13.6 (10.6, 16.7) million children under 5 developed SAM (6)" [lines 57-58]

20. Also consider citing the increasing evidence (e.g. two recent systematic reviews in BMJ Global Health) about the long term adverse effects of childhood SAM. This would also be worth including in the discussion as better treatment might have long term benefits: hence the costs of treatment might in fact have much more long lasting benefit and be even more cost-effective than appears in short-term analysis such as this.

Authors' response: We have edited the introduction and discussion sections to include this material. Edited text in the introduction section shown here: "Children surviving SAM experience higher rates of morbidity, mortality, and other adverse outcomes later in life (4, 5)" [lines 56-57]

Edited text in the discussion section shown here: "Recent systematic reviews (4, 5) have demonstrated poorer long-term outcomes among children surviving SAM compared to those without a history of SAM, suggesting that more effective SAM treatment may ameliorate these long-term effects." [lines 482-484]

21. Lines 90-95. Did participants attend until 12 weeks irrespective of growth/progress or could they be discharged earlier if meeting specified anthro criteria (e.g. WLZ >-2 z-score on 2 consecutive visits – as is standard in many CMAM programmes worldwide. Please give details for both arms as again this could make a big difference to final costs.

Authors' response: We thank the reviewer for highlighting this point of confusion and confirm that children were discharged from the nutritional treatment program when they met the criteria for recovery, defined in both groups as being free from medical complications, having MUAC ≥ 125 mm on two consecutive clinic visits, and no edema for a maximum of 12 weeks (lines 181-183).

22. Line 100-107. Please summarise sample size rationale/justification. What outcomes was the trial powered to be able to observe? Did power calcs consider economic outcomes and if so what assumptions were made in the sample size calcs for this?

Authors' response: We have added summary information about sample size calculations, and referred to the primary paper reporting the primary effectiveness endpoints which provides greater detail: "The target sample size in each study arm (1750, with 175 per OTP) was calculated to achieve >90% power to detect a noninferiority margin difference between the group proportions of −0.10. A detailed description of trial design and sample size calculations are provided in Hitchings et al (15)." [lines 113-116]

23. Line 156 – assuming this is an economic analysis of a main trial reported elsewhere, re-phrase this section to make this clear. Consider also including a summary of the main trial results here.

Authors' response: We confirm that this is an economic analysis of a main trial reported elsewhere, as suggested by the reviewer. We have edited this section to clarify that the major trial outcomes are reported elsewhere, and have included primary effectiveness endpoints as a supplementary table: "Table S1 provides summary results for major effectiveness endpoints, as reported in Hitchings et al (15)." [lines 189-191]

24. Line 158 – good to mention these discharge criteria earlier as per previous comment. Was discharges only based on MUAC or was weight-for-length recovery also considered?

Authors' response: We confirm that discharge as recovered (primary outcome of the main trial) was defined as per the study protocol as being free from medical complications, having MUAC ≥ 125 mm on two consecutive clinic visits, and no edema at or before 12 weeks. Please refer to lines 181-183.

25. Even though this is an econ evaluation, a study flow chart would be helpful in first para of results – to get a sense of completeness of data and balance of drop-out/default between study arms.

Authors' response: Thank you for this suggestion, we have added a study flow chart, and text briefly describing it: "There were 3788 children enrolled in the clinical study with a recorded outcome at program discharge (1802 in the standard-of-care arm, 1976 in the monthly follow-up arm) and 3594 children with an outcome recorded at study discharge at 3 months post-program discharge (1721 in standard-of-care, 1873 in monthly follow-up). Of these children, 1031 were included in the outpatient costing sub-study and 686 in the inpatient costing sub-study. Additional details are provided in Figure 1." [lines 239-244]

26. Would be good if the main effectiveness results from the main paper are summed up previously. But if not, should do so here in the first para rather than much further down.

Authors' response: We have moved the relevant section to the start of the results section. This section is intended to summarize the primary health outcomes used for the cost-effectiveness analysis, and therefore does not include all outcomes of the effectiveness evaluation, and also includes an outcome (DALYs) not included in the other paper. We have retitled this section for clarity "Effectiveness outcomes for cost-effectiveness analysis" (line 239). We have included the main study endpoints from the primary effectiveness paper as a supplementary table (Table S1), reproduced from Hitchings et al 2022 (line 190-191).

27. Line 211 – please also mention average in-programme stay (in weeks) in this section...this would help put the number of consults into context.

Authors' response: As suggested by the reviewer, we have added results on the average time to recovery (reported in the primary analysis, see Hitchings et al) to the results section: "Among children who recovered, the time between enrollment and program discharge was greater in the monthly follow-up group compared to standard-of-care (67.4 days vs. 51.2 days, p<0.001)." [lines 261-263]

28. Line 217 – rather than "non-significant" better to just cite the p-value.

Authors' response: We have edited these instances to provide the p-values.

29. Line 276 - % recovery seems very low in both arms – important to discuss this later in discussion. Was for example % default very high? A table summarising all standard CMAM effectiveness outcomes would be helpful (perhaps as online annex so readers don't have to search for the main paper, ref 14).

Authors' response: We agree with the reviewer that the primary health outcomes of the main trial may be of interest. We have edited and extended the text in the discussion section to include more representation of the primary health outcomes, which are all more thoroughly discussed in the primary paper (see Hitchings et al): "Firstly, while these results provide evidence against the monthly follow-up approach as a replacement for standard-of-care in this setting, they do not describe how these results might generalize to other settings. In particular, the health outcomes observed for nutritional recovery under both arms in this study were below international standards (38) and may be influenced by contextual factors including poor access to care or suboptimal home use of RUTF (as discussed by Hitchings et al (15)), which may vary across settings. It is unclear how the cost-effectiveness outcomes reported in this study would change in settings with a higher fraction of children achieving nutritional recovery." [lines 470-471]

We have also included the primary effectiveness endpoints as a supplementary table: "Table S1 provides summary results for major effectiveness endpoints, as reported in Hitchings et al (15)." [lines 190-191]

30. Why was a monthly follow-up chosen? It's quite a big jump from weekly to monthly – with hindsight (and for future research, esp in light of the observation re RUTF accounting for the greater costs) perhaps 2 or 3weekly follow-up would have been a better comparator against the "standard" weekly follow-up. Add to discussion/recommendation for future research.

Authors' response: Monthly follow-up (as opposed to biweekly or every 3 weeks) was chosen after discussion with the study's scientific advisors. In the contexts where this strategy might be useful (e.g. difficult geographic access, high insecurity, and high burden), visits every 2 were not considered to offer a meaningful difference for programs or participants. As such, a monthly program was considered and deemed to be safe and feasible following pilot evaluation in Niger. Further, we note that biweekly is already standard care in some settings (for example, in Malawi), such that evaluating biweekly visits would not be of minimal scientific contribution. Text

discussing alternative visit timing is now included in the Discussion, at the reviewer's recommendation (edited text shown under reviewer comment below).

31. Shorten conclusions section to be clearer what the study found. Do this by putting some of the text currently there into a "recommendations for future research" section in the prior discussion.

Authors' response: Thank you for this suggestion, we have moved the discussion of alternative future approaches to the discussion section: "<u>Alternative approaches should remain under consideration to reduce caregiver burden while promoting clinical recovery</u>. Possible modifications could include adopting a visit frequency between the approaches tested in this trial (such as weekly follow-up until initial weight gain is achieved followed by monthly follow-up until discharge), basing the frequency of follow-up on an initial clinical risk assessment, or allowing caregivers to play a greater role in choosing the frequency of follow-up given full information of the various trade-offs. It is possible that these or other modifications could improve efficiency without jeopardizing health outcomes." [lines 454-463]
